# Development and validation of a nomogram for predicting hospitalization-associated disability in older patients with acute heart failure

**DOI:** 10.3389/fcvm.2026.1770434

**Published:** 2026-02-27

**Authors:** Xiaonan Hao, Fei Li, Yan Gu, Huijing Zhang, Xinyu Chen, Kun Li

**Affiliations:** 1 School of Nursing, Jilin University, Changchun, China; 2 Department of Endocrinology and Metabolism, The First Hospital of Jilin University, Changchun, China; 3 Department of Cardiovascular Disease Center, The First Hospital of Jilin University, Changchun, China

**Keywords:** activities of daily living, disability, heart failure, hospitalization, nomogram, older adults

## Abstract

**Objectives:**

To develop and validate a nomogram for predicting hospitalization-associated disability in older patients with acute heart failure.

**Design:**

A single-center cohort study.

**Setting and participants:**

This study was carried out in the Cardiovascular Disease Center of a large tertiary-care hospital in China. Between December 2024 and February 2025, 480 older patients with acute heart failure were enrolled.

**Methods:**

Hospitalization-associated disability was defined as a decline of 5 points or more in the Barthel Index from admission to discharge. Predictor screening involved univariable logistic regression, Spearman's correlation, and Least Absolute Shrinkage and Selection Operator regression. Variables retained were entered into a multivariable logistic regression model, and significant predictors were used to construct a nomogram for predicting hospitalization-associated disability. Model performance was assessed in terms of discrimination, calibration, and clinical utility.

**Results:**

The incidence of hospitalization-associated disability was 41.88%. A 12-variable nomogram was developed, incorporating age, ejection fraction, emergency admission, comorbidity burden, cognitive function, nutritional risk, pre-admission activities of daily living, pre-admission instrumental activities of daily living, physical mobility, sleep disturbance, depressive symptoms, and perceived social support. The nomogram demonstrated robust discrimination, with the area under the receiver operating characteristic curve of 0.841 and 0.786 in the training and testing sets, respectively. Calibration was accurate in both sets. The training set achieved a mean absolute error of 0.037 and a Brier score of 0.154, while the testing set showed 0.026 and 0.188, accompanied by non-significant Hosmer-Lemeshow test results. Decision curve and clinical impact analyses further supported favorable clinical utility.

**Conclusions and implications:**

A 12-variable nomogram was developed and validated in older individuals with acute heart failure, enabling early identification of hospitalization-associated disability risk and supporting personalized care strategies.

## 1 Introduction

Heart failure (HF) represents a growing public health challenge in the 21st century. Its prevalence increases progressively with age, and HF remains the leading cause of hospitalization among individuals aged 65 years and older (1, 2). While acute hemodynamic instability can be effectively managed with conventional therapies, hospitalization-associated disability (HAD), a multifactorial condition resulting from acute illness, patient vulnerabilities, and hospital care processes, may substantially compromise long-term prognosis (3). HAD is defined as a decline in activities of daily living (ADLs) occurring between hospital admission and discharge in older adults (4, 5). A Japanese multicenter cohort study reported a high HAD incidence of 37.1% among older patients hospitalized with HF (6), and further evidence indicates that HAD independently predicts HF-related readmission and all-cause mortality (7). Accordingly, early identification and proactive prevention of HAD are critical to improving prognosis in this population.

Developing clinical prediction models is essential for the early identification of HAD risk among older inpatients. Existing tools, such as the HAD-FREE Score, integrate seven predictors to estimate HAD risk in general medical inpatients (8). However, accumulating evidence suggests that trajectories of functional decline vary substantially across disease categories. A Swedish cohort study demonstrated distinct functional deterioration patterns in patients with cardiovascular diseases compared with those with other medical conditions (9), indicating that non-specific models may have limited predictive performance in HF populations. Moreover, current tools insufficiently capture the multidimensional stressors emphasized in Chang's Nosocomial Based Stress Model, including adverse emotional responses and environmental factors inherent to hospitalization, which can impede mental well-being and functional recovery (10). Therefore, both a multidimensional risk assessment framework and the dynamic tracking of ADLs from pre- to post-hospitalization are warranted to develop an HF-specific HAD prediction model and improve the precision of high-risk patient identification.

The nomogram, as an intuitive and user-friendly predictive tool, offers significant advantages in clinical research and practice. Primarily, it can transform complex multifactorial prediction models into visual representations, enabling clinicians to efficiently and accurately assess individual patients' risk probabilities (11). Furthermore, nomograms exhibit excellent interpretability and practicality, facilitating patient-provider communication and improving patient comprehension of disease risks and treatment plans, thereby fostering greater adherence (12).

This research focused on constructing and validating a predictive nomogram to assess HAD risk in older individuals diagnosed with acute HF. The clinical value of this predictive instrument lies in its ability to facilitate the early identification of vulnerable patient subgroups and subsequently guide personalized care protocols, thereby effectively reducing the risk of HAD development.

## 2 Methods

### 2.1 Study design and setting

The research was conducted as a prospective, single-center cohort study between December 2024 and February 2025 at the Cardiovascular Disease Center of a tertiary-care hospital in China. The study was conducted and reported following the TRIPOD statement, which guides transparent reporting of multivariable prediction models for prognosis or diagnosis (13).

### 2.2 Study participants

A convenience sampling method was employed to enroll older patients with acute HF. The standards for including participants in this research were as follows: (1) HF as the primary reason for hospitalization; (2) admission to the general wards; (3) age ≥ 60 years; and (4) provision of informed consent. The exclusion standards were as follows: (1) discharge from the hospital within 48 h; (2) complete dependence in ADLs, which precludes the further functional decline required for developing HAD; (3) significant hearing, visual, or communication impairments; and (4) a life expectancy of less than one month.

### 2.3 Sample size calculation

The minimum sample size was calculated using the framework by Riley et al. for multivariable prediction models with binary outcomes (14). Assumptions included a 30% HAD incidence based on a meta-analysis of older patients (15), and an area under the receiver operating characteristic curve (AUC) of 0.87 for model discrimination (16). Accordingly, the Cox-Snell *R*^2^ was estimated to be 0.34 using the approach described in the methodological literature (17). Furthermore, a total of 12 predictors were assumed, including 8 variables reported in a study of general medical inpatients (18) and 4 additional variables intended to capture HF-specific characteristics and potential psychosocial indicators during hospitalization. This resulted in a minimum sample size of 323, deemed sufficient to ensure robust model development with adequate calibration and generalizability.

### 2.4 Definition of HAD

HAD was defined as a reduction of 5 points or more in a patient's capacity to carry out ADLs, as measured by the Barthel Index (BI) at admission and discharge intervals. The BI, a well-established 10-item measurement scale, evaluates functional independence across two critical dimensions: self-care and mobility competencies (19). The BI categorizes patients into the following clinical dependency levels: complete independence, which is scored as 100; mild dependence, corresponding to scores ranging from 61 to 99; moderate dependence, with scores falling between 41 and 60; and severe dependence, indicated by scores of 40 or less.

### 2.5 Predictive variables

#### 2.5.1 Demographic characteristics

Demographic data were collected at enrollment through structured interviews, including age, sex, educational attainment, marital status, place of residence, living arrangement, monthly household income, and payment source.

#### 2.5.2 Clinical and behavioral characteristics

Clinical and behavioral characteristics were obtained from electronic medical records and structured interviews. Clinical variables included laboratory indicators such as serum creatinine, hemoglobin, fasting blood glucose, and total cholesterol, as well as cardiac function measures including ejection fraction (EF) and New York Heart Association (NYHA) Class. Additional clinical information comprised length of hospital stay, route of admission and cause of HF. Behavioral characteristics included smoking history and alcohol consumption.

#### 2.5.3 Functional status

##### 2.5.3.1 Baseline physiological functional reserve

Baseline functional status was comprehensively evaluated during the initial 24 h of hospitalization. Pre-admission ADLs two weeks prior to hospitalization was assessed retrospectively using the BI, while instrumental ADLs (IADLs) were assessed using the Lawton-Brody scale (score range: 0–8, higher scores indicating greater independence) (20). Comorbidity burden was quantified using the Charlson Comorbidity Index (CCI) (21), with patients categorized as having low (1–2), moderate (3–4), or high (≥5) comorbidity burden. Cognitive function was measured utilizing the Mini-Mental State Examination (MMSE) (22), with scores categorized as normal cognition (≥27), mild impairment (21–26), and moderate-to-severe impairment (≤20). Nutritional risk was assessed using the Malnutrition Universal Screening Tool (MUST) (23), and patients were categorized as having low (0), moderate (1), or high risk (2 or more) of malnutrition according to established criteria.

##### 2.5.3.2 Hospitalization-related variables

Hospitalization-related functional variables were assessed after the first 48 h of admission to capture patients' physical, psychological, and social status during the inpatient care phase. Physical mobility were quantified using the rating of mobility (24), with scores ranging from complete bedrest (0) to independent ambulation at least twice daily (12 points). Sleep disturbances were measured using the Insomnia Severity Index (ISI) (25), with scores categorized as no clinically significant insomnia (≤7), mild-to-moderate insomnia (8–21), and severe insomnia (≥22). Psychological distress was assessed through the Hospital Anxiety and Depression Scale (HADS) (26), including anxiety (HADS-A) and depressive (HADS-D) subscales. A subscale score exceeding 7 indicates the presence of clinically meaningful symptoms. Perceived Social Support during hospitalization was examined using the standardized Medical Outcomes Study Social Support Survey (MOS-SSS) (27), with total scores ranging from 19 to 95, where higher scores indicate greater perceived social support.

### 2.6 Statistical analysis

Data analysis was conducted within the R statistical environment (version 4.4.2) (28). Since missingness was limited to covariates and affected merely 6 patients (1.23%), a rate below 5%, a complete-case analysis was appropriately employed (29, 30). Descriptive statistics summarized categorical and continuous variables. Categorical data were analyzed using frequency distributions and chi-square tests. Continuous data were assessed for normality using the Shapiro–Wilk test, with *P*-values below 0.05 indicating non-normality. Since all continuous variables were non-normally distributed, medians (IQRs) were reported, and between-group differences were evaluated with the Mann–Whitney *U*-test.

Participants were randomly assigned to training and testing sets in a 7:3 ratio using simple random sampling (31). A multi-stage variable screening strategy was employed to select strong predictors for nomogram construction, with HAD serving as the dependent variable throughout the entire process. The process began with univariable logistic regression to assess the association between each candidate variable and HAD, retaining variables with *P* < 0.05 for further analysis. To address potential multicollinearity among continuous variables, Spearman's rank correlation was used (32). Any variable pairs with a correlation coefficient exceeding the pre-defined threshold of 0.8 would be reviewed, with the variable deemed to have lesser clinical relevance or weaker univariable statistical importance being removed. Remaining variables underwent Least Absolute Shrinkage and Selection Operator (LASSO) regression for feature selection, with the optimal *λ* determined via 10-fold cross-validation (33). Variables with non-zero coefficients in the LASSO model were then included in a multivariable logistic regression model, where associations were quantified as odds ratios (ORs) with 95% confidence intervals (CIs). The final predictors for the nomogram were those with statistically significant associations in the multivariable model.

The nomogram's performance was assessed in terms of discrimination, calibration, and clinical utility. Discrimination was evaluated using receiver operating characteristic curve analysis, with the AUC as the primary indicator; an AUC > 0.75 indicated satisfactory discrimination (34). Calibration was evaluated using three approaches. Calibration curves visually assessed the agreement between predicted probabilities and observed outcomes. The Hosmer-Lemeshow test assessed goodness-of-fit, with a non-significant result indicating adequate fit. The Brier score quantified overall prediction accuracy, with a score below 0.25 indicating good calibration (35). Clinical utility was evaluated through decision curve analysis (DCA), which quantified net benefit across various threshold probabilities. Additionally, a clinical impact curve (CIC) was constructed to illustrate the number of high-risk individuals classified and true positives identified at each threshold.

### 2.7 Ethical considerations

Approval for this research protocol was granted by the Ethics Committee of the School of Nursing, Jilin University (No. 2024120301). Prior to enrollment, written informed consent was obtained from all participants.

## 3 Results

### 3.1 Participant characteristics

Table 1 summarizes the demographic, clinical, behavioral, and functional characteristics of the 480 participants. The cohort had a median age of 75.50 years, with females comprising 56.88%. Most patients (72.92%) had HF secondary to ischemic or hypertensive heart disease, and the median length of hospital stay was 9 days. By discharge, 201 participants developed HAD, yielding an incidence of 41.88%. Participants were randomly assigned to training (*n* = 336) and testing (*n* = 144) cohorts, which differed significantly in educational attainment (*P* = 0.040), living arrangements (*P* = 0.031), and rating of mobility (*P* = 0.047). Comparison between HAD and non-HAD groups revealed significant differences in demographic, clinical and functional characteristics, such as age, EF, route of admission, and scores on the IADLs scale (see Appendix Supplementary Table S1).

**Table 1:** Differences in characteristics between the training cohort and the testing cohort.

Variables	Total (*N* = 480) M (Q1, Q3)/*n* (%)	Training cohort (*N* = 336) M (Q1, Q3)/*n* (%)	Testing cohort (*N* = 144) M (Q1, Q3)/*n* (%)	*P*-value
Demographic characteristics				
Age (years)				0.796^a^
60–70	162 (33.75)	116 (34.52)	46 (31.94)	
71–80	161 (33.54)	113 (33.63)	48 (33.33)	
≥81	157 (32.71)	107 (31.85)	50 (34.72)	
Sex				0.533^a^
Male	207 (43.12)	148 (44.05)	59 (40.97)	
Female	273 (56.88)	188 (55.95)	85 (59.03)	
Educational attainment				**0**.**040**^a^
Illiteracy	144 (30.00)	92 (27.38)	52 (36.11)	
Primary school	183 (38.12)	124 (36.90)	59 (40.97)	
Junior high school	107 (22.29)	83 (24.70)	24 (16.67)	
Senior high school and above	46 (9.58)	37 (11.01)	9 (6.25)	
Marital status				0.557^a^
Single	39 (8.12)	30 (8.93)	9 (6.25)	
Married	310 (64.58)	213 (63.39)	97 (67.36)	
Divorced	46 (9.58)	35 (10.42)	11 (7.64)	
Widowed	85 (17.71)	58 (17.26)	27 (18.75)	
Place of residence				0.149^a^
Urban	170 (35.42)	110 (32.74)	60 (41.67)	
Township	158 (32.92)	113 (33.63)	45 (31.25)	
Rural	152 (31.67)	113 (33.63)	39 (27.08)	
Living arrangement				**0**.**031**^a^
Living Alone	103 (21.46)	81 (24.11)	22 (15.28)	
Living with Spouse/Children	377 (78.54)	255 (75.89)	122 (84.72)	
Family income (yuan/month)				0.577^a^
≤1,000	128 (26.67)	91 (27.08)	37 (25.69)	
1,001–3,000	173 (36.04)	115 (34.23)	58 (40.28)	
3,001–5,000	101 (21.04)	75 (22.32)	26 (18.06)	
≥5,001	78 (16.25)	55 (16.37)	23 (15.97)	
Payment Source				0.663^a^
Employee Health Insurance	63 (13.12)	46 (13.69)	17 (11.81)	
Urban Resident Health Insurance	121 (25.21)	86 (25.60)	35 (24.31)	
New Rural Cooperative Health Insurance	134 (27.92)	87 (25.89)	47 (32.64)	
Out-of-pocket	139 (28.96)	100 (29.76)	39 (27.08)	
Commercial Insurance	23 (4.79)	17 (5.06)	6 (4.17)	
Clinical and behavioral characteristics				
Length of hospital stay (days)	9.00 (9.00, 10.00)	9.00 (9.00, 10.00)	9.00 (9.00,10.00)	0.769^b^
BMI (kg/m^2^)	25.10 (22.28, 27.90)	24.90 (22.17, 27.80)	25.45 (22.60, 28.72)	0.159^b^
SBP (mmHg)	132.00 (115.75, 150.00)	132.00 (114.00, 151.25)	134.50 (119.00, 150.00)	0.395^b^
DBP (mmHg)	82.00 (78.00, 86.00)	82.00 (78.00, 86.00)	82.00 (79.00, 86.00)	0.252^b^
HR (bmp)	92.00 (78.00, 106.00)	91.00 (77.00, 107.00)	93.00 (83.00, 105.00)	0.271^b^
BNP (pg/mL)	1,667.50 (1,034.00, 2,344.75)	1,636.50 (1,001.25, 2,336.50)	1,697.50 (1,100.25, 2,366.00)	0.444^b^
Hb (g/L)	146.00 (134.00, 155.00)	146.00 (134.00, 155.00)	146.00 (130.25, 155.00)	0.899^b^
FBG (mmol/L)	5.20 (4.90, 5.60)	5.20 (4.90, 5.60)	5.20 (4.90, 5.62)	0.839^b^
Na^+^ (mmol/L)	141.90 (140.80, 143.10)	141.95 (140.97, 143.10)	141.85 (140.50, 142.95)	0.300^b^
K^+^(mmol/L)	4.42 (4.17, 4.64)	4.43 (4.18, 4.64)	4.42 (4.13, 4.63)	0.967^b^
SUA (*μ*mol/L)	338.00 (301.75, 446.00)	338.50 (303.00, 439.00)	335.50 (297.75, 449.75)	0.657^b^
TC (mmol/L)	4.86 (3.79, 6.20)	4.84 (3.87, 6.16)	4.91 (3.55, 6.20)	0.737^b^
SCr (μmol/L)				0.750^a^
≤110	238 (49.58)	165 (49.11)	73 (50.69)	
>111	242 (50.42)	171 (50.89)	71 (49.31)	
NYHA Class				0.524^a^
Class III	246 (51.25)	169 (50.30)	77 (53.47)	
Class IV	234 (48.75)	167 (49.70)	67 (46.53)	
EF (%)				0.335^a^
≥61	163 (33.96)	121 (36.01)	42 (29.17)	
41–60	158 (32.92)	106 (31.55)	52 (36.11)	
≤40	159 (33.12)	109 (32.44)	50 (34.72)	
*De novo* HF				0.232^a^
Yes	250 (52.08)	181 (53.87)	69 (47.92)	
No	230 (47.92)	155 (46.13)	75 (52.08)	
Route of admission				0.690^a^
Elective admission	230 (47.92)	163 (48.51)	67 (46.53)	
Emergency admission	250 (52.08)	173 (51.49)	77 (53.47)	
Cause of HF				0.866^a^
Ischemic heart disease	186 (38.75)	126 (37.50)	60 (41.67)	
Hypertensive heart disease	164 (34.17)	116 (34.52)	48 (33.33)	
Valvular heart disease	67 (13.96)	48 (14.29)	19 (13.19)	
Dilated cardiomyopathy	34 (7.08)	26 (7.74)	8 (5.56)	
Others	29 (6.04)	20 (5.95)	9 (6.25)	
Smoking status				0.261^a^
Never smoker	269 (56.04)	185 (55.06)	84 (58.33)	
Former smoker	127 (26.46)	86 (25.60)	41 (28.47)	
Current smoker	84 (17.50)	65 (19.35)	19 (13.19)	
Alcohol use				0.190^a^
Never drinker	314 (65.42)	217 (64.58)	97 (67.36)	
Former drinker	88 (18.33)	58 (17.26)	30 (20.83)	
Current drinker	78 (16.25)	61 (18.15)	17 (11.81)	
Functional status				
Rating of mobility	6.00 (4.00, 8.00)	6.00 (4.00, 8.00)	5.00 (4.00, 6.00)	**0**.**047**^b^
IADLs	5.00 (2.75, 6.00)	4.00 (2.00, 6.00)	5.00 (3.00, 6.00)	0.055^b^
MOS-SSS	50.00 (46.00, 56.00)	51.00 (46.00, 56.00)	49.0 (45.00, 55.00)	0.171^b^
Admission BI	55.00 (40.00, 85.00)	55.00 (40.00, 85.00)	55.00 (40.00, 86.25)	0.204^b^
Discharge BI	50.00 (35.00, 80.00)	50.00 (35.00, 85.00)	55.00 (40.00, 80.00)	0.271^b^
HAD				0.455^a^
Yes	201 (41.88)	137 (40.77)	64 (44.44)	
No	279 (58.12)	199 (59.23)	80 (55.56)	
CCI				0.404^a^
1–2	152 (31.67)	111 (33.04)	41 (28.47)	
3–4	163 (33.96)	108 (32.14)	55 (38.19)	
≥5	165 (34.38)	117 (34.82)	48 (33.33)	
MMSE				0.571^a^
≥27	158 (32.92)	112 (33.33)	46 (31.94)	
21–26	167 (34.79)	112 (33.33)	55 (38.19)	
≤20	155 (32.29)	112 (33.33)	43 (29.86)	
MUST				0.825^a^
0	166 (34.58)	118 (35.12)	48 (33.33)	
1	157 (32.71)	107 (31.85)	50 (34.72)	
≥2	157 (32.71)	111 (33.04)	46 (31.94)	
Pre-admission BI				0.616^a^
≥61	169 (35.21)	114 (33.93)	55 (38.19)	
41–60	148 (30.83)	104 (30.95)	44 (30.56)	
≤40	163 (33.96)	118 (35.12)	45 (31.25)	
ISI				0.148^a^
≤7	160 (33.33)	121 (36.01)	39 (27.08)	
8–21	168 (35.00)	111 (33.04)	57 (39.58)	
≥22	152 (31.67)	104 (30.95)	48 (33.33)	
HADS-A				0.076^a^
≤7	223 (46.46)	165 (49.11)	58 (40.28)	
>7	257 (53.54)	171 (50.89)	86 (59.72)	
HADS-D				0.437^a^
≤7	253 (52.71)	181 (53.87)	72 (50.00)	
>7	227 (47.29)	155 (46.13)	72 (50.00)	

Bold data indicates *P* < 0.05.

BI, barthel index; BMI, body mass index; BNP, B-type natriuretic peptide; CCI, charlson comorbidity index; DBP, diastolic blood pressure; EF, ejection fraction; FBG, fasting blood glucose; HAD, hospitalization-associated disability; HADS-A, hospital anxiety and depression scale-anxiety; HADS-D, hospital anxiety and depression scale-depression; Hb, hemoglobin; HF, heart failure; HR, heart rate; IADLs, instrumental activities of daily living; ISI, insomnia severity index; MMSE, mini-mental state examination; MOS-SSS, medical outcomes study social support survey; MUST, malnutrition universal screening tool; NYHA class, New York heart association functional classification; SBP, systolic blood pressure; SCr, serum creatinine; SUA, serum uric acid; TC, total cholesterol.

^a^ Indicates chi-square test.

^b^ Indicates Mann–Whitney *U*-test.

### 3.2 Predictor screening

#### 3.2.1 Univariable logistic regression analysis

A comprehensive literature review and theory-based rationale identified 39 candidate predictors, which were then analyzed via univariable logistic regression. Thirteen significant variables associated with HAD were identified (see Appendix Supplementary Table S2). These included age 81 years or older, urban resident health insurance, emergency admission, EF of 40% or less, high comorbidity burden (CCI ≥ 5), severe pre-admission functional dependency (BI ≤ 40), insomnia (ISI ≥ 8), moderate to severe cognitive impairment (MMSE ≤ 20), nutritional risk (MUST ≥ 1), depressive symptoms (HADS-D > 7), and scores on rating of mobility, IADLs scale, and MOS-SSS.

#### 3.2.2 Bivariate correlation analysis

The results of the Spearman correlation analysis, presented in Supplementary Table S3 (see Appendix), indicated that all correlation coefficients between variable pairs were well below the pre-defined threshold of 0.8. Furthermore, all *P*-values were greater than 0.05, indicating an absence of statistically significant correlations. Consequently, no variables were excluded.

#### 3.2.3 LASSO regression analysis

**Figure 1:**
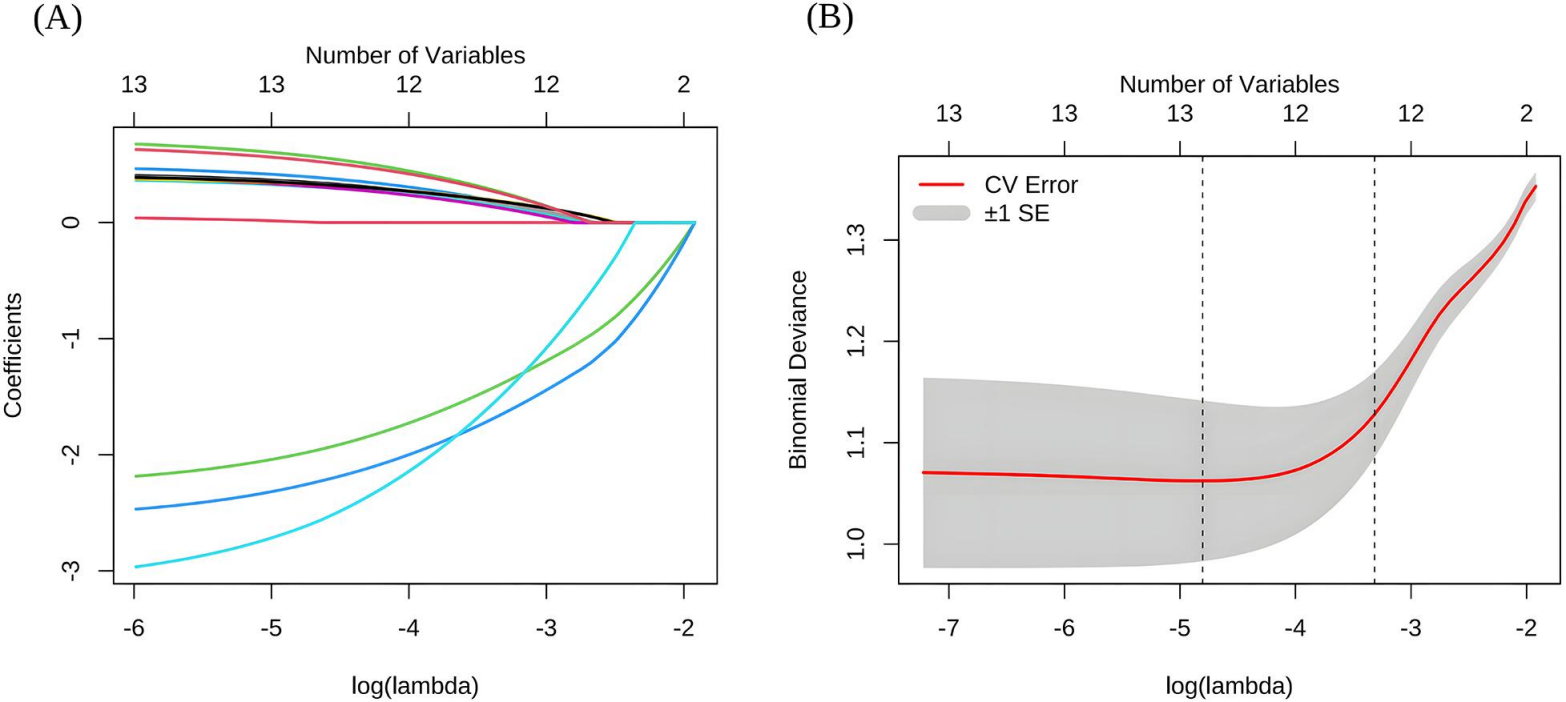
Selection of optimal features using LASSO regression. **(A)** Coefficient trajectories of features across different log(λ) values. **(B)** Cross-validation curve for selecting the optimal λ value in the LASSO regression model. CV, cross-validation; LASSO, least absolute shrinkage and selection operator; SE, standard error.

To refine the selection of candidate variables and mitigate potential overfitting, LASSO regression was applied to the pool of 13 variables retained from the univariable screening and correlation analysis. The model selection process is illustrated in Figure 1. Specifically, the coefficient profiles for all variables are shown in Figure 1A, which displays the coefficient trajectories for candidate predictors across different values of log(λ), illustrating the progressive shrinkage of coefficients toward zero as λ increases. The optimal λ was simultaneously identified via 10-fold cross-validation (Figure 1B), where the mean binomial deviance (cross-validation error) is plotted against log(λ), with a shaded area representing standard errors. Two critical λ values were identified: λ.min (λ = 0.008), corresponding to the minimum cross-validation error (left dashed line), and λ.1SE (λ = 0.036), defined as the largest λ for which the cross-validation error remains within one standard error of the minimum (right dashed line). For model finalization, λ.1SE was selected, as it resulted in a model with fewer predictors, favoring simplicity while preserving predictive performance. Ultimately, 12 variables were screened, including age, route of admission, EF, as well as scores on the CCI, MMSE, MUST, ISI, HADS-D, pre-admission BI, IADLs scale, rating of mobility, and MOS-SSS.

### 3.3 Prediction model development

The 12 variables filtered out through LASSO regression method were integrated into a multivariate logistic regression analysis for further modeling. Risk factors indicating increased odds of HAD included age 81 years or older (OR = 2.34, 95 % CI = 1.175–4.663, *P* = 0.016), emergency admission (OR = 2.01, 95 % CI = 1.146–3.535, *P* = 0.015), EF of 40% or less (OR = 2.78, 95 % CI = 1.390–5.549, *P* = 0.004), high comorbidity burden (OR = 2.11, 95 % CI = 1.057–4.213, *P* = 0.034), mild cognitive impairment (OR = 2.05, 95 % CI = 1.017–4.115, *P* = 0.045), moderate to severe cognitive impairment (OR = 2.26, 95 % CI = 1.132–4.507, *P* = 0.021), high nutritional risk (OR = 2.20, 95 % CI = 1.119–4.336, *P* = 0.022), severe pre-admission functional dependence (OR = 2.30, 95 % CI = 1.153–4.568, *P* = 0.018), severe insomnia (OR = 2.36, 95 % CI = 1.185–4.700, *P* = 0.015), and depressive symptoms (OR = 1.92, 95 % CI = 1.093–3.380, *P* = 0.023). Conversely, protective factors associated with significantly decreased odds of HAD were higher scores on the rating of mobility (OR = 0.81, 95 % CI = 0.732–0.891, *P* < 0.001), the IADLs scale (OR = 0.72, 95 % CI = 0.626–0.818, *P* < 0.001), and the MOS-SSS (OR = 0.94, 95 % CI = 0.907–0.969, *P* < 0.001). Further details are presented in Table 2.

**Table 2:** Multivariate logistic regression analysis of predictors for HAD.

Variables	*β*	SE	*Z*-value	OR (95% CI)	*P*-value
Age (years)					
60–70	Ref				
71–80	0.314	0.345	0.910	1.37 (0.696–2.694)	0.363
≥81	0.851	0.352	2.420	2.34 (1.175–4.663)	**0**.**016**
Route of admission					
Elective admission	Ref				
Emergency admission	0.699	0.287	2.433	2.01 (1.146–3.535)	**0**.**015**
EF (%)					
≥61	Ref				
41–60	0.486	0.345	1.408	1.63 (0.827–3.195)	0.159
≤40	1.022	0.353	2.893	2.78 (1.390–5.549)	**0**.**004**
CCI					
1–2	Ref				
3–4	0.256	0.350	0.732	1.29 (0.651–2.566)	0.464
≥5	0.747	0.353	2.118	2.11 (1.057–4.213)	**0**.**034**
MMSE					
≥27	Ref				
21–26	0.716	0.357	2.007	2.05 (1.017–4.115)	**0**.**045**
≤20	0.815	0.352	2.312	2.26 (1.132–4.507)	**0**.**021**
MUST					
0	Ref				
1	0.376	0.353	1.067	1.46 (0.730–2.907)	0.286
≥2	0.790	0.346	2.286	2.20 (1.119–4.336)	**0**.**022**
Pre-admission BI					
≥61	Ref				
41–60	0.452	0.354	1.278	1.57 (0.786–3.145)	0.201
≤40	0.831	0.351	2.366	2.30 (1.153–4.568)	**0**.**018**
ISI					
≤7	Ref				
8–21	0.659	0.347	1.898	1.93 (0.979–3.818)	0.058
≥22	0.859	0.352	2.443	2.36 (1.185–4.700)	**0**.**015**
HADS-D					
≤7	Ref				
>7	0.653	0.288	2.270	1.92 (1.093–3.380)	**0**.**023**
Rating of mobility	−0.214	0.050	−4.260	0.81 (0.732–0.891)	**<0**.**001**
IADLs	−0.334	0.068	−4.904	0.72 (0.626–0.818)	**<0**.**001**
MOS-SSS	−0.065	0.017	−3.825	0.94 (0.907–0.969)	**<0**.**001**

Bold data indicates *P* < 0.05.

BI, barthel index; CCI, charlson comorbidity index; CI, confidence interval; EF, ejection fraction; HAD, hospitalization-associated disability; HADS-D, hospital anxiety and depression scale-depression; IADLs, instrumental activities of daily living; ISI, insomnia severity index; MMSE, mini-mental state examination; MOS-SSS, medical outcomes study social support survey; MUST, malnutrition universal screening tool; SE, standard error.

**Figure 2:**
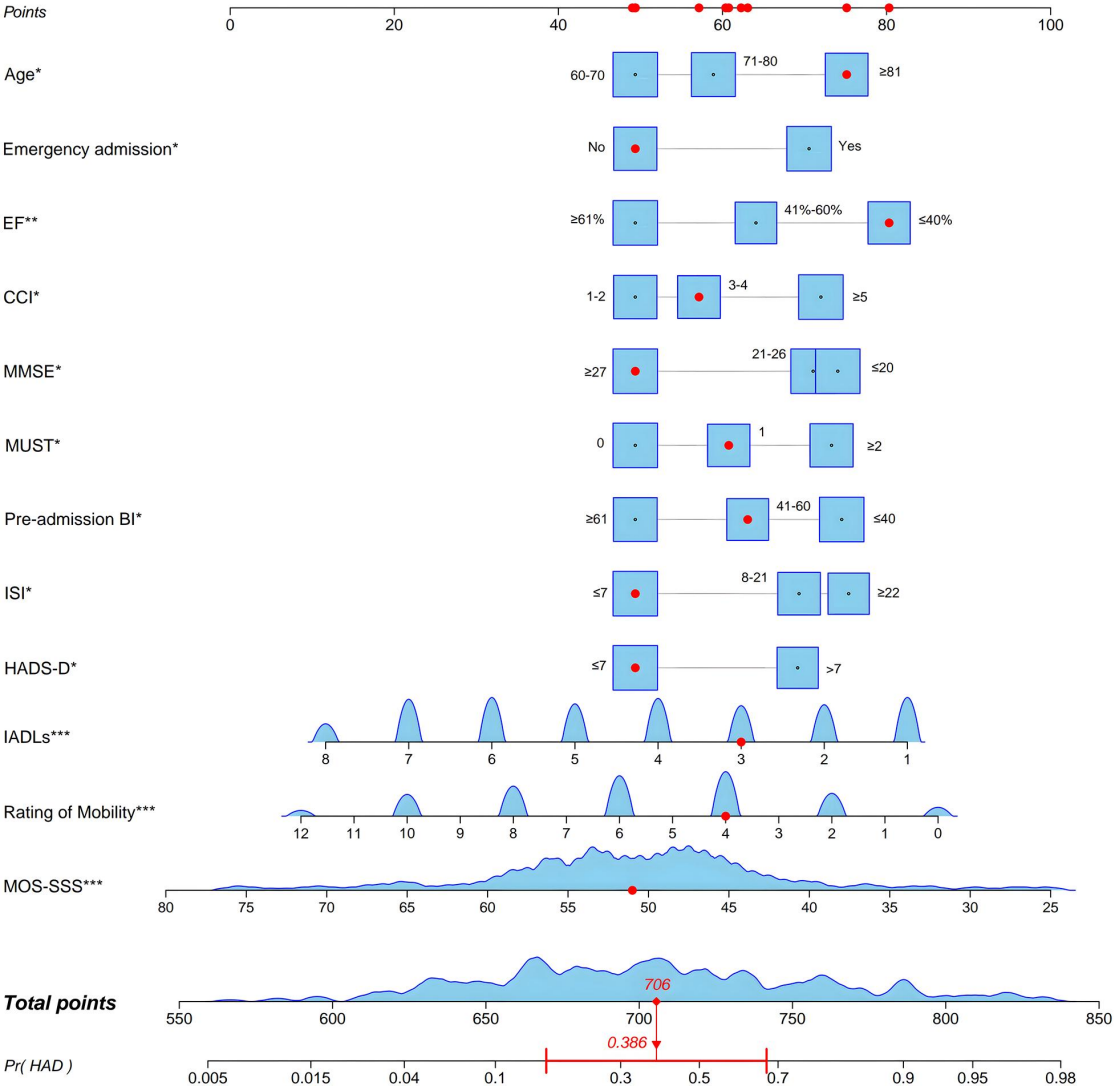
Nomogram for predicting the probability of HAD in older patients with acute HF. For example, consider a patient with the following characteristics: aged ≥81 years; elective admission; with an EF ≤40%; a CCI score of 3–4; a score of 3 on the IADLs Scale; an MMSE score ≥27; a pre-admission BI between 41 and 60; an ISI score ≤7; a HADS–D score ≤7; a mobility rating of 4; and a MOS–SSS score of 51. The corresponding points for each variable were obtained by locating the red dots on the top “Points” axis of the nomogram. These individual points were then summed to yield a total point of approximately 706. According to the bottom “Pr (HAD)” axis, this total point corresponds to a 0.386 probability of HAD occurrence. BI, barthel index; CCI, charlson comorbidity index; EF, ejection fraction; HAD, hospitalization-associated disability; HADS-D, hospital anxiety and depression scale-depression; HF, heart failure; IADLs, instrumental activities of daily living; ISI, insomnia severity index; MMSE, mini-mental state examination; MOS-SSS, medical outcomes study social support survey.

Guided by the multivariable logistic regression findings, a predictive nomogram incorporating 12 variables was developed to estimate HAD risk among older individuals diagnosed with acute HF (Figure 2). When utilizing the nomogram, the patient's scores for each variable should first be identified, and the corresponding points are then obtained by locating the red dots on the top “Points” axis. Subsequently, these points are added together to calculate the total point. Finally, a vertical projection from the “Total points” axis to the “Pr (HAD)” axis provides an estimate of the probability of HAD. For instance, the total point for the 15th patient was 706, corresponding to an estimated 38.6% probability of developing HAD.

**Figure 3:**
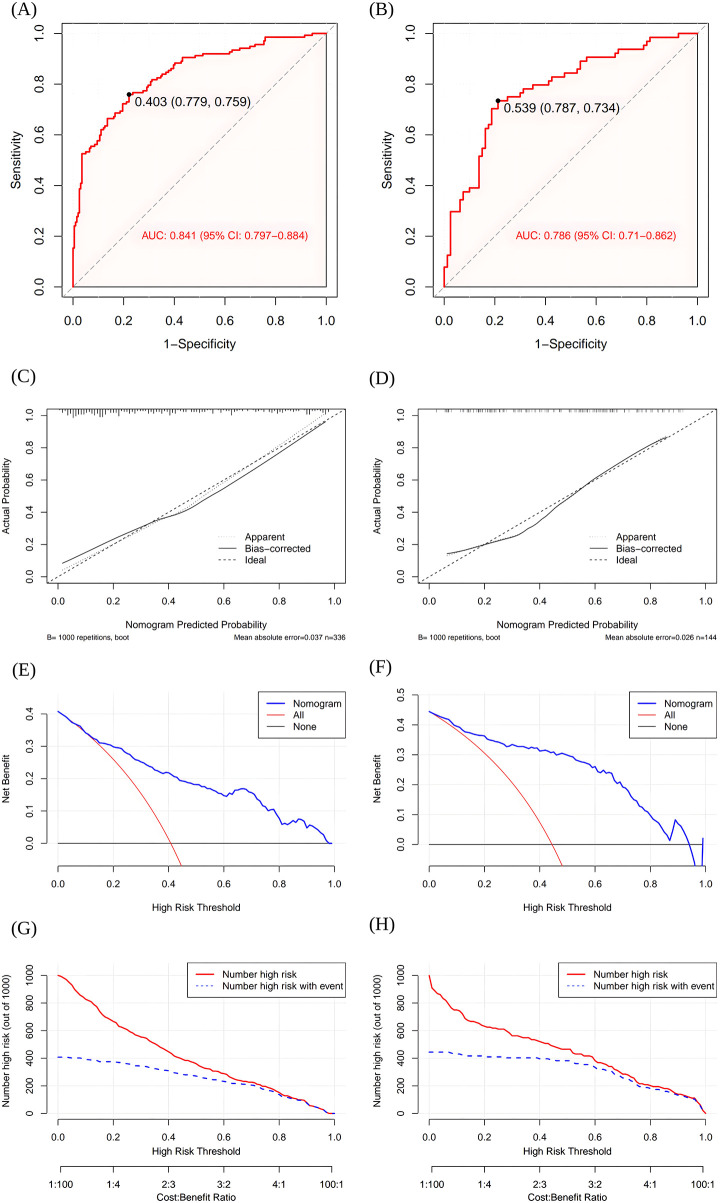
Performance assessment of the nomogram. **(A)** Receiver operating characteristic curve of the nomogram in the training group. **(B)** Receiver operating characteristic curve of the nomogram in the testing group. **(C)** Calibration curve of the nomogram in the training group. **(D)** Calibration curve of the nomogram in the testing group. **(E)** Decision curve analysis of the nomogram in the training group. **(F)** Decision curve analysis of the nomogram in the testing group. **(G)** Clinical impact curve of the nomogram in the training group. **(H)** Clinical impact curve of the nomogram in the testing group.

The nomogram demonstrated good discrimination, with an AUC of 0.841 (95% CI = 0.797–0.884) (Figure 3A). Calibration analysis indicated strong agreement between predicted and observed HAD probabilities (mean absolute erro*r* = 0.037; Figure 3C). This was further supported by a non-significant Hosmer-Lemeshow test (*χ*^2^ = 7.75, *P* = 0.459) and a Brier score of 0.155, reinforcing the model's good calibration. Moreover, DCA revealed that across most of the threshold probability range (0–1), the nomogram provided a superior net benefit compared with two extreme clinical strategies illustrated in Figure 3E, namely the “All” strategy represented by the red line, which involves treating all individuals regardless of their risk, and the “None” strategy represented by the black line, which involves treating no individuals. The CIC graphically reinforced this utility (Figure 3G). It demonstrated that, particularly when the threshold probability exceeded 0.6, the number of individuals identified as high-risk closely matched actual HAD cases. These results support the nomogram's robust performance and clinical utility.

### 3.4 Prediction model testing

Internal validation through a 30% stratified random subset demonstrated the nomogram's robust predictive performance. The AUC for the testing cohort was 0.786 (95% CI = 0.710–0.862) (Figure 3B). The calibration plot illustrated a remarkable consistency between the estimated probabilities of HAD and the actual ones, quantified by a mean absolute error of 0.026 (Figure 3D). This strong calibration was confirmed by a non-significant Hosmer-Lemeshow test result (*χ*² = 6.85, *P* = 0.553) and supported by a Brier score of 0.188. Furthermore, based on the DCA, the nomogram provided a higher net benefit across a broad range of threshold probabilities, outperforming both the “All” strategy (red line) and the “None” strategy (black line) (Figure 3F). The CIC analysis corroborated this finding, showing that at high-risk thresholds above 0.6, the number of predicted cases closely aligned with the actual number of events (Figure 3H). Together, these findings support the favorable clinical utility of the nomogram for guiding HAD prediction and intervention decisions.

## 4 Discussion

This study developed and validated a nomogram to predict HAD in older patients with acute HF, filling an important gap in prediction models for this population. The nomogram integrating 12 variables demonstrated robust performance in discrimination, calibration, and clinical utility. By enabling the early identification of high-risk individuals, it serves as a practical tool to guide clinical decision-making and facilitate timely interventions aimed at reducing the risk of HAD development.

### 4.1 Analysis of HAD incidence

The incidence of HAD in older Chinese patients with acute HF was 41.88%, higher than the 37.1% reported in a recent Japanese multicenter cohort study (6). This difference can be explained by several factors. First, the median EF in our cohort was 43.00%, notably below the 48.0% reported in the Japanese study, while the median pre-admission BI was 60.00, significantly lower than their 88.3. These differences indicate that patients in this study experienced more severe cardiac dysfunction and possessed a poorer baseline physiological functional reserve, both of which significantly increased the likelihood of developing HAD, as demonstrated by the constructed nomogram. Furthermore, variations in inpatient management practices may also contribute to this disparity. Participants in the Japanese study engaged in standardized inpatient cardiac rehabilitation protocols, incorporating early low-intensity mobilization strategies, progressive endurance exercises, and structured resistance training regimens. This individualized, multicomponent strategy may have contributed to shorter bed-rest times, fostering functional recovery and thereby mitigating the risk of HAD.

### 4.2 Analysis of HAD predictors

Reduced EF and emergency admission, which were identified as independent predictors of HAD through multivariable logistic regression, were subsequently visualized in the nomogram to serve as indicators of disease severity. A novel contribution of the present study is the emphasis on the important role of cardiac function in the development of HAD. An EF of 40% or less denotes a marked decline in cardiac output, driving chronic hypoperfusion that accelerates skeletal muscle ischemia and loss (36). Although direct quantification of muscle strength or mass was not performed, prior studies reported a 2.5-fold higher sarcopenia risk among patients with reduced EF compared with those with preserved EF (37), implying a diminished muscular reserve in the reduced-EF subgroup. Recent evidence also indicates that HF patients with preserved EF are at a similarly high risk of developing HAD. In populations primarily characterized by this phenotype, factors such as advanced age, a high burden of comorbidities, significant frailty, and nutritional risks contribute to a heightened baseline vulnerability to functional decline (38). These findings suggest that the mechanisms leading to HAD may vary depending on the phenotype. In patients with reduced EF, the risk is predominantly driven by cardiac dysfunction and its systemic effects, whereas in those with preserved EF, the risk is more closely linked to broader vulnerabilities, such as geriatric syndromes, multimorbidity, and impaired physical capacity. Emergency admission represents an acute dimension of risk. Consistent with our findings, prior geriatric research demonstrated that emergency admission approximately doubled the odds of HAD compared with elective admission (8). This association may be explained by the sudden worsening of HF symptoms that triggers a cascade of stress responses such as neuroendocrine dysregulation and immune imbalance (39), which hinder recovery and accelerate functional decline, as described in the Nosocomial Based Stress Model (10). Furthermore, urgent therapeutic measures (e.g., diuretics for pulmonary edema) may induce electrolyte shifts such as hypokalemia, resulting in fatigue and dizziness that limit patient mobility and exacerbate in-hospital muscle deconditioning (40).

The nomogram integrated six critical variables reflecting baseline physiological functional reserve, including age, comorbidity burden, cognitive function, nutritional risk, and pre-admission functional status in ADLs and IADLs. Among these variables, cognitive impairment emerged as a particularly influential predictor, wherein even mild deficits significantly increased the risk of HAD. This finding aligns with prior studies focusing on older cardiovascular patients, which established cognitive impairment as an independent predictor of HAD (41, 42). In particular, Honda et al. reported that cognitive impairment, as a univariate predictor, achieved an AUC of 0.77, demonstrating its discriminative power and critical role in HAD development (42). Nutritional risk was also retained, consistent with earlier work that identified loss of appetite as a predictor in cardiac patients (41). A noteworthy study suggested that the CONUT score, an objective indicator derived from serum albumin, total cholesterol, and total lymphocyte count, may offer superior predictive value for HAD in older patients with acute HF compared to tools relying on self-reported items, highlighting the potential utility of biomarker-based nutritional assessments in this population (43). The remaining predictors, including advanced age, multiple comorbidities, pre-admission functional dependence in ADLs and IADLs, have all been established as statistically significant predictors of HAD in previous cohort studies (44–46). Overall, these results support the multidimensional cumulative impairment paradigm, in which co-occurring deficits across physiological systems synergistically compromise adaptive capacity and heighten vulnerability to functional decline following acute illness or iatrogenic stressors (3).

The nomogram further incorporated four variables that reflect functional status during hospitalization, including physical mobility, sleep disturbance, depressive symptoms, and perceived social support. Among these, physical mobility, typically reflected by low movement levels, emerges as one of the most prevalent deficits (47). In line with our findings, prior studies have consistently demonstrated that in-hospital immobility increases the risk of HAD regardless of the mobility metric employed (8, 46, 48, 49). Furthermore, this association has been quantitatively examined in patients with acute HF, where each additional day of delayed ambulation was independently associated with a 17% increase in the odds of impaired ADLs at discharge (50). According to current Chinese guidelines, an early mobilization protocol, comprising low-intensity resistance training, joint mobilization, and respiratory muscle training, should be initiated as soon as vital signs stabilize (51). However, in real-world clinical settings, traditional “bed-rest” culture, concerns about fall risk, and overprotective attitudes among family members often lead to a substantial gap between recommended and actual levels of mobility. Moreover, by integrating sleep disturbance, depressive symptoms, and perceived social support into a HAD prediction framework for older patients with acute HF, this study provides novel validation of the Nosocomial Based Stress Model in this cohort. Specifically, the effect sizes of these psychosocial stressors were comparable to those of established physiological indices within the multivariable model, highlighting their significant role in functional deterioration. According to the Nosocomial Based Stress Model, the mechanistic explanation for this finding is that inpatient psychosocial stressors interact synergistically with illness-induced physiological responses, amplifying neuroendocrine and immune dysfunction, while concurrently fostering a hypervigilant cognitive state that undermines rehabilitation engagement and accelerates functional decline (10). Therefore, routinely assessing these psychosocial stressors in clinical practice not only refines high-risk identification but also provides more actionable targets for preventive interventions.

### 4.3 Implications for theory and practice

This study advances both the theoretical understanding and clinical practice of HAD through the development and validation of a predictive nomogram. From a theoretical perspective, although our prior systematic review categorized HAD risk factors into baseline vulnerability (e.g., frailty) and in-hospital process factors (e.g., immobility), the role of psychosocial stressors during hospitalization has remained underexplored (52). Guided by the Nosocomial Based Stress Model, this research demonstrates that depressive symptoms and low perceived social support serve as independent predictors of HAD, thus validating and extending the applicability of this model in older adults with acute HF.

From a practical standpoint, the nomogram integrates three complementary risk dimensions, including baseline vulnerability, acute disease severity during hospitalization, and modifiable in-hospital stressors. This integration addresses a critical limitation of existing frailty assessments, which primarily capture pre-admission deficits, by enabling more accurate identification of high-risk patients, including those with relatively preserved baseline function. Consequently, the nomogram provides empirical support for a multidimensional risk framework of HAD and serves as a valuable complement to conventional clinical assessments, facilitating early, targeted preventive interventions tailored to specific risk profiles.

### 4.4 Limitations

Several limitations should be acknowledged. First, the single-center design and convenience sampling approach has the potential to introduce selection bias, restricting the applicability of the study results to other healthcare settings or populations. Second, although the selection of a broad range of predictors was guided by literature review and theoretical rationale, it remains inherently subjective. Third, external validation for the developed nomogram was not implemented in the current research, which may introduce uncertainties regarding its performance. Fourth, while the nomogram integrates routinely assessed clinical variables, applying it at the bedside may pose practical challenges in fast-paced clinical environments without the support of a dedicated electronic tool. Despite these limitations, this study represents a meaningful step toward precise estimation of HAD risk in geriatric HF care. Future research should prioritize multicenter collaborations, integrate objective biomarkers, validate the nomogram in diverse ethnic and healthcare contexts, and focus on the development of integrated digital tools (e.g., clinical calculators or mobile applications) to facilitate point-of-care risk assessment and enhance clinical adoption, thereby further refining predictive accuracy, scientific validity, and practical applicability.

## 5 Conclusion

In this research, an innovative prediction model was developed and validated for predicting HAD specifically within older individuals experiencing acute HF. The model exhibited strong discriminatory power and reliable calibration, offering clinicians an intuitive instrument to identify high-risk individuals in the early stages. The findings highlight the multifactorial nature of HAD, revealing the interplay among disease severity, baseline physiological reserve deficits, and hospitalization-induced stressors. Furthermore, the nomogram supports targeted, risk-informed interventions during hospitalization, with the aim of mitigating functional decline and preserving independence in older patients with acute HF.

## Data Availability

The raw data supporting the conclusions of this article will be made available by the authors, without undue reservation.
