# Supplementary material for: Development and validation of a nomogram for predicting hospitalization-associated disability in older patients with acute heart failure
Source: Front Cardiovasc Med. 2026 Feb 27;13:1770434. doi: 10.3389/fcvm.2026.1770434 (PMC12982025; doi:10.3389/fcvm.2026.1770434)
Supplement: Supplementary file 1 [file Datasheet1.docx]

**Catalogue**

1. **Table S1.** Differences in characteristics between the HAD cohort and the non-HAD cohort.
2. **Table S2.** Univariable logistic regression analysis of predictors for HAD.
3. **Table S3.** Spearman’s correlation matrix for continuous variables retained from the univariable analysis.

| **Table S1.** Differences in characteristics between the HAD cohort and the non-HAD cohort. | | | |
| --- | --- | --- | --- |
| Variables | HAD (*N*=201)  M (Q1, Q3)/n (%) | Non-HAD (*N*=279)  M (Q1, Q3)/n (%) | *P*-value |
| **Demographic characteristics** |  |  |  |
| Age (years) |  |  | **0.006** ^a^ |
| 60-70 | 53 (26.37) | 109 (39.07) |  |
| 71-80 | 69 (34.33) | 92 (32.97) |  |
| ≥81 | 79 (39.30) | 78 (27.96) |  |
| Sex |  |  | 0.753 ^a^ |
| Male | 85 (42.29) | 122 (43.73) |  |
| Female | 116 (57.71) | 157 (56.27) |  |
| Educational attainment |  |  | 0.951 ^a^ |
| Illiteracy | 63 (31.34) | 81 (29.03) |  |
| Primary school | 76 (37.81) | 107 (38.35) |  |
| Junior high school | 43 (21.39) | 64 (22.94) |  |
| Senior high school and above | 19 (9.45) | 27 (9.68) |  |
| Marital status |  |  | 0.453 ^a^ |
| Single | 14 (6.97) | 25 (8.96) |  |
| Married | 138 (68.66) | 172 (61.65) |  |
| Divorced | 18 (8.96) | 28 (10.04) |  |
| Widowed | 31 (15.42) | 54 (19.35) |  |
| Place of residence |  |  | 0.819 ^a^ |
| Urban | 71 (35.32) | 99 (35.48) |  |
| Township | 69 (34.33) | 89 (31.90) |  |
| Rural | 61 (30.35) | 91 (32.62) |  |
| Living arrangement |  |  | 0.976 ^a^ |
| Living Alone | 43 (21.39) | 60 (21.51) |  |
| Living with Spouse/Children | 158 (78.61) | 219 (78.49) |  |
| Family income (yuan/month) |  |  | 0.652 ^a^ |
| ≤1000 | 54 (26.87) | 74 (26.52) |  |
| 1001-3000 | 78 (38.81) | 95 (34.05) |  |
| 3001-5000 | 38 (18.91) | 63 (22.58) |  |
| ≥5001 | 31 (15.42) | 47 (16.85) |  |
| Payment Source |  |  | 0.241 ^a^ |
| Employee Health Insurance | 32 (15.92) | 31 (11.11) |  |
| Urban Resident Health Insurance | 45 (22.39) | 76 (27.24) |  |
| New Rural Cooperative Health Insurance | 56 (27.86) | 78 (27.96) |  |
| Out-of-pocket | 55 (27.36) | 84 (30.11) |  |
| Commercial Insurance | 13 (6.47) | 10 (3.58) |  |
| **Clinical and Behavioral Characteristics** |  |  |  |
| Length of hospital stay (days) | 9.00 (9.00, 10.00) | 9.00 (9.00, 10.00) | 0.214 ^b^ |
| BMI (kg/m²) | 24.90 (22.20, 27.90) | 25.30 (22.40, 27.90) | 0.505 ^b^ |
| SBP (mmHg) | 131.00 (116.00, 150.00) | 133.00 (115.00, 152.00) | 0.772 ^b^ |
| DBP (mmHg) | 82.00 (78.00, 86.00) | 82.00 (78.50, 86.00) | 0.740 ^b^ |
| HR (bmp) | 91.00 (78.00, 106.00) | 93.00 (78.00, 105.00) | 0.880 ^b^ |

| **Table S1.** (continued). | | | |
| --- | --- | --- | --- |
| Variables | HAD (*N*=201)  M (Q1, Q3)/n (%) | Non-HAD (*N*=279)  M (Q1, Q3)/n (%) | *P*-value |
| BNP (pg/mL) | 1717.00 (1006.00, 2335.00) | 1659.00 (1046.00, 2351.50) | 0.888 ^b^ |
| Hb (g/L) | 143.00 (128.00, 155.00) | 147.00 (136.50, 155.00) | 0.076 ^b^ |
| FBG (mmol/L) | 5.30 (4.90, 5.80) | 5.20 (4.90, 5.60) | 0.191 ^b^ |
| Na⁺ (mmol/L) | 141.90 (140.80, 143.00) | 142.00 (140.90, 143.10) | 0.402 ^b^ |
| K⁺(mmol/L) | 4.45 (4.14, 4.64) | 4.40 (4.18, 4.63) | 0.866 ^b^ |
| SUA (μmol/L) | 340.00 (302.00, 445.00) | 337.00 (301.50, 446.00) | 0.803 ^b^ |
| TC (mmol/L) | 4.85 (3.80, 6.19) | 4.89 (3.79, 6.20) | 0.614 ^b^ |
| SCr (μmol/L) |  |  | **0.048** ^a^ |
| ≤110 | 89 (44.28) | 149 (53.41) |  |
| >111 | 112 (55.72) | 130 (46.59) |  |
| NYHA Class |  |  | **0.042** ^a^ |
| Class III | 92 (45.77) | 154 (55.20) |  |
| Class IV | 109 (54.23) | 125 (44.80) |  |
| EF (%) |  |  | **0.001** ^a^ |
| ≥61 | 51 (25.37) | 112 (40.14) |  |
| 41-60 | 69 (34.33) | 89 (31.90) |  |
| ≤40 | 81 (40.30) | 78 (27.96) |  |
| De novo HF |  |  | 0.385 ^a^ |
| Yes | 100 (49.75) | 150 (53.76) |  |
| No | 101 (50.25) | 129 (46.24) |  |
| Route of admission |  |  | **<0.001** ^a^ |
| Elective admission | 78 (38.81) | 152 (54.48) |  |
| Emergency admission | 123 (61.19) | 127 (45.52) |  |
| Cause of HF |  |  | 0.226 ^a^ |
| Ischemic heart disease | 88 (43.78) | 98 (35.13) |  |
| Hypertensive heart disease | 59 (29.35) | 105 (37.63) |  |
| Valvular heart disease | 25 (12.44) | 42 (15.05) |  |
| Dilated cardiomyopathy | 16 (7.96) | 18 (6.45) |  |
| Others | 13 (6.47) | 16 (5.73) |  |
| Smoking status |  |  | 0.955 ^a^ |
| Never smoker | 113 (56.22) | 156 (55.91) |  |
| Former smoker | 54 (26.87) | 73 (26.16) |  |
| Current smoker | 34 (16.92) | 50 (17.92) |  |
| Alcohol use |  |  | 0.164 ^a^ |
| Never drinker | 139 (69.15) | 175 (62.72) |  |
| Former drinker | 29 (14.43) | 59 (21.15) |  |
| Current drinker | 33 (16.42) | 45 (16.13) |  |
| **Functional status** |  |  |  |
| Rating of mobility | 4.00 (2.00, 6.00) | 6.00 (4.00, 8.00) | **<0.001** ^b^ |
| IADLs | 4.00 (2.00, 6.00) | 5.00 (3.00, 7.00) | **<0.001** ^b^ |
| MOS-SSS | 47.00 (44.00, 52.00) | 53.00 (48.00, 56.00) | **<0.001** ^b^ |

| **Table S1.** (continued). | | | |
| --- | --- | --- | --- |
| Variables | HAD (*N*=201)  M (Q1, Q3)/n (%) | Non-HAD (*N*=279)  M (Q1, Q3)/n (%) | *P*-value |
| Admission BI | 50.00 (40.00, 85.00) | 55.00 (40.00, 85.00) | 0.104 ^b^ |
| Discharge BI | 40.00 (25.00, 70.00) | 60.00 (40.00, 90.00) | **<0.001** ^b^ |
| CCI |  |  | **0.006** ^a^ |
| 1-2 | 48 (23.88) | 104 (37.28) |  |
| 3-4 | 73 (36.32) | 90 (32.26) |  |
| ≥5 | 80 (39.80) | 85 (30.47) |  |
| MMSE |  |  | **0.011** ^a^ |
| ≥27 | 51 (25.37) | 107 (38.35) |  |
| 21-26 | 76 (37.81) | 91 (32.62) |  |
| ≤20 | 74 (36.82) | 81 (29.03) |  |
| MUST |  |  | **0.034** ^a^ |
| 0 | 57 (28.36) | 109 (39.07) |  |
| 1 | 68 (33.83) | 89 (31.90) |  |
| ≥2 | 76 (37.81) | 81 (29.03) |  |
| Pre-admission BI |  |  | 0.070 ^a^ |
| ≥61 | 59 (29.35) | 110 (39.43) |  |
| 41-60 | 66 (32.84) | 82 (29.39) |  |
| ≤40 | 76 (37.81) | 87 (31.18) |  |
| ISI |  |  | **<0.001** ^a^ |
| ≤7 | 48 (23.88) | 112 (40.14) |  |
| 8-21 | 77 (38.31) | 91 (32.62) |  |
| ≥22 | 76 (37.81) | 76 (27.24) |  |
| HADS-A |  |  | 0.171 ^a^ |
| ≤7 | 86 (42.79) | 137 (49.10) |  |
| >7 | 115 (57.21) | 142 (50.90) |  |
| HADS-D |  |  | **0.010** ^a^ |
| ≤7 | 92 (45.77) | 161 (57.71) |  |
| >7 | 109 (54.23) | 118 (42.29) |  |

Note:

Bold data indicates *P* < 0.05; ^a^ indicates chi-square test; ^b^ indicates Mann-Whitney *U* test

Abbreviations: BI = Barthel Index; BMI = Body Mass Index; BNP = B-type Natriuretic Peptide; CCI = Charlson Comorbidity Index; DBP = Diastolic Blood Pressure; EF = Ejection Fraction; FBG = Fasting Blood Glucose; HAD = Hospitalization-associated Disability; HADS-A = Hospital Anxiety and Depression Scale-Anxiety; HADS-D = Hospital Anxiety and Depression Scale-Depression; Hb = Hemoglobin; HF = Heart Failure; HR = Heart Rate; IADLs = Instrumental Activities of Daily Living; ISI = Insomnia Severity Index; MMSE = Mini-Mental State Examination; MOS-SSS = Medical Outcomes Study Social Support Survey; MUST = Malnutrition Universal Screening Tool; NYHA Class = New York Heart Association Functional Classification; SBP = Systolic Blood Pressure; SCr = Serum Creatinine; SUA = Serum Uric Acid; TC = Total Cholesterol

| **Table S2.** Univariable logistic regression analysis of predictors for HAD. | | | | | |
| --- | --- | --- | --- | --- | --- |
| Variables | *β* | SE | Z-value | OR (95%CI) | *P*-value |
| **Demographic characteristics** |  |  |  |  |  |
| Age (years) |  |  |  |  |  |
| 60-70 | Ref |  |  |  |  |
| 71-80 | 0.230 | 0.275 | 0.836 | 1.26 (0.734-2.160) | 0.403 |
| ≥81 | 0.699 | 0.276 | 2.535 | 2.01 (1.172-3.453) | **0.011** |
| Sex |  |  |  |  |  |
| Male | Ref |  |  |  |  |
| Female | -0.083 | 0.223 | -0.370 | 0.92 (0.594-1.427) | 0.711 |
| Educational attainment |  |  |  |  |  |
| Illiteracy | Ref |  |  |  |  |
| Primary school | -0.142 | 0.281 | -0.506 | 0.87 (0.500-1.505) | 0.613 |
| Junior high school | 0.085 | 0.306 | 0.277 | 1.09 (0.597-1.984) | 0.782 |
| Senior high school and above | 0.079 | 0.394 | 0.202 | 1.08 (0.501-2.342) | 0.840 |
| Marital status | Ref |  |  |  |  |
| Single | 0.330 | 0.403 | 0.818 | 1.39 (0.631-3.065) | 0.413 |
| Married | -0.234 | 0.525 | -0.445 | 0.79 (0.283-2.217) | 0.657 |
| Divorced | -0.095 | 0.469 | -0.203 | 0.91 (0.363-2.279) | 0.839 |
| Place of residence |  |  |  |  |  |
| Urban | Ref |  |  |  |  |
| Township | -0.267 | 0.271 | -0.985 | 0.77 (0.450-1.302) | 0.324 |
| Rural | -0.417 | 0.274 | -1.526 | 0.66 (0.385-1.126) | 0.127 |
| Living arrangement |  |  |  |  |  |
| Living Alone | Ref |  |  |  |  |
| Living with Spouse/Children | 0.138 | 0.262 | 0.526 | 1.15 (0.687-1.916) | 0.599 |
| Family income (yuan/month) |  |  |  |  |  |
| ≤1000 | Ref |  |  |  |  |
| 1001-3000 | 0.131 | 0.283 | 0.463 | 1.14 (0.655-1.983) | 0.643 |
| 3001-5000 | -0.405 | 0.324 | -1.252 | 0.67 (0.353-1.258) | 0.211 |
| ≥5001 | -0.272 | 0.351 | -0.774 | 0.76 (0.383-1.517) | 0.439 |
| Payment Source |  |  |  |  |  |
| Employee Health Insurance | Ref |  |  |  |  |
| Urban Resident Health Insurance | -0.782 | 0.375 | -2.082 | 0.46 (0.219-0.955) | **0.037** |
| New Rural Cooperative Health Insurance | -0.208 | 0.365 | -0.568 | 0.81 (0.397-1.662) | 0.570 |
| Out-of-pocket | -0.447 | 0.359 | -1.245 | 0.64 (0.316-1.293) | 0.213 |
| Commercial Insurance | 0.118 | 0.568 | 0.207 | 1.12 (0.369-3.427) | 0.836 |
| **Clinical and Behavioral Characteristics** |  |  |  |  |  |
| Length of hospital stay (days) | 0.032 | 0.115 | 0.274 | 1.03 (0.823-1.293) | 0.784 |
| BMI (kg/m²) | -0.033 | 0.031 | -1.043 | 0.97 (0.910-1.029) | 0.297 |
| SBP (mmHg) | 0.000 | 0.004 | 0.095 | 1.00 (0.992-1.009) | 0.924 |
| DBP (mmHg) | -0.024 | 0.016 | -1.467 | 0.98 (0.946-1.008) | 0.142 |
| HR (bmp) | 0.005 | 0.006 | 0.794 | 1.01 (0.993-1.016) | 0.427 |

| **Table S2.** (continued). | | | | | |
| --- | --- | --- | --- | --- | --- |
| Variables | *β* | SE | Z-value | OR (95% CI) | *P*-value |
| BNP (pg/mL) | 0.000 | 0.000 | 0.714 | 1.00 (1.000-1.000) | 0.475 |
| Hb (g/L) | -0.007 | 0.007 | -0.955 | 0.99 (0.979-1.007) | 0.340 |
| FBG (mmol/L) | 0.070 | 0.065 | 1.078 | 1.07 (0.944-1.218) | 0.281 |
| Na⁺ (mmol/L) | -0.026 | 0.044 | -0.578 | 0.98 (0.894-1.063) | 0.564 |
| K⁺(mmol/L) | 0.266 | 0.269 | 0.988 | 1.31 (0.770-2.213) | 0.323 |
| SUA (μmol/L) | -0.000 | 0.001 | -0.250 | 1.00 (0.997-1.002) | 0.802 |
| TC (mmol/L) | 0.027 | 0.080 | 0.345 | 1.03 (0.879-1.202) | 0.730 |
| SCr (μmol/L) |  |  |  |  |  |
| ≤110 | Ref |  |  |  |  |
| >111 | 0.162 | 0.222 | 0.727 | 1.18 (0.760-1.818) | 0.467 |
| NYHA Class |  |  |  |  |  |
| Class III | Ref |  |  |  |  |
| Class IV | 0.341 | 0.223 | 1.532 | 1.41 (0.909-2.178) | 0.126 |
| EF (%) |  |  |  |  |  |
| ≥61 | Ref |  |  |  |  |
| 41-60 | 0.363 | 0.276 | 1.314 | 1.44 (0.837-2.469) | 0.189 |
| ≤40 | 0.651 | 0.272 | 2.390 | 1.92 (1.124-3.267) | **0.017** |
| De novo HF |  |  |  |  |  |
| Yes | Ref |  |  |  |  |
| No | -0.060 | 0.223 | -0.267 | 0.94 (0.609-1.458) | 0.789 |
| Route of admission |  |  |  |  |  |
| Elective admission | Ref |  |  |  |  |
| Emergency admission | 0.672 | 0.226 | 2.974 | 1.96 (1.258-3.051) | **0.003** |
| Cause of HF |  |  |  |  |  |
| Ischemic heart disease | Ref |  |  |  |  |
| Hypertensive heart disease | -0.306 | 0.263 | -1.165 | 0.74 (0.440-1.233) | 0.244 |
| Valvular heart disease | -0.200 | 0.345 | -0.578 | 0.82 (0.416-1.611) | 0.563 |
| Dilated cardiomyopathy | -0.087 | 0.436 | -0.200 | 0.92 (0.390-2.153) | 0.842 |
| Others | -0.182 | 0.490 | -0.372 | 0.83 (0.319-2.179) | 0.710 |
| Smoking status |  |  |  |  |  |
| Never smoker | Ref |  |  |  |  |
| Former smoker | -0.301 | 0.269 | -1.120 | 0.74 (0.436-1.254) | 0.263 |
| Current smoker | -0.134 | 0.293 | -0.455 | 0.88 (0.492-1.555) | 0.649 |
| Alcohol use |  |  |  |  |  |
| Never drinker | Ref |  |  |  |  |
| Former drinker | -0.316 | 0.309 | -1.025 | 0.73 (0.398-1.334) | 0.305 |
| Current drinker | 0.028 | 0.293 | 0.096 | 1.03 (0.579-1.827) | 0.923 |
| **Functional status** |  |  |  |  |  |
| Rating of mobility | -0.223 | 0.042 | -5.255 | 0.80 (0.736-0.869) | **< 0.001** |
| IADLs | -0.295 | 0.056 | -5.308 | 0.75 (0.668-0.830) | **< 0.001** |
| MOS-SSS | -0.049 | 0.014 | -3.539 | 0.95 (0.927-0.979) | **< 0.001** |

| **Table S2.** (continued). | | | | | |
| --- | --- | --- | --- | --- | --- |
| Variables | *β* | SE | Z-value | OR (95%CI) | *P*-value |
| CCI |  |  |  |  |  |
| 1-2 | Ref |  |  |  |  |
| 3-4 | 0.477 | 0.282 | 1.690 | 1.61 (0.927-2.801) | 0.091 |
| ≥5 | 0.690 | 0.276 | 2.503 | 1.99 (1.161-3.422) | **0.012** |
| MMSE |  |  |  |  |  |
| ≥27 | Ref |  |  |  |  |
| 21-26 | 0.537 | 0.279 | 1.925 | 1.71 (0.990-2.956) | 0.054 |
| ≤20 | 0.681 | 0.278 | 2.449 | 1.98 (1.146-3.409) | **0.014** |
| MUST |  |  |  |  |  |
| 0 | Ref |  |  |  |  |
| 1 | 0.702 | 0.283 | 2.482 | 2.02 (1.159-3.513) | **0.013** |
| ≥2 | 1.000 | 0.280 | 3.578 | 2.72 (1.572-4.702) | **< 0.001** |
| Pre-admission BI |  |  |  |  |  |
| ≥61 | Ref |  |  |  |  |
| 41-60 | 0.504 | 0.284 | 1.775 | 1.66 (0.949-2.888) | 0.076 |
| ≤40 | 0.780 | 0.274 | 2.846 | 2.18 (1.275-3.734) | **0.004** |
| ISI |  |  |  |  |  |
| ≤7 | Ref |  |  |  |  |
| 8-21 | 0.741 | 0.278 | 2.664 | 2.10 (1.216-3.617) | **0.008** |
| ≥22 | 0.978 | 0.282 | 3.471 | 2.66 (1.531-4.619) | **< 0.001** |
| HADS-A |  |  |  |  |  |
| ≤7 | Ref |  |  |  |  |
| >7 | 0.410 | 0.224 | 1.834 | 1.51 (0.972-2.335) | 0.067 |
| HADS-D |  |  |  |  |  |
| ≤7 | Ref |  |  |  |  |
| >7 | 0.638 | 0.225 | 2.837 | 1.89 (1.218-2.940) | **0.005** |

Note:

Bold data indicates *P* < 0.05.

Abbreviations: BI = Barthel Index; BMI = Body Mass Index; BNP = B-type Natriuretic Peptide; CCI = Charlson Comorbidity Index; CI = Confidence Interval; DBP = Diastolic Blood Pressure; EF = Ejection Fraction; FBG = Fasting Blood Glucose; HAD = Hospitalization-associated Disability; HADS-A = Hospital Anxiety and Depression Scale-Anxiety; HADS-D = Hospital Anxiety and Depression Scale-Depression; Hb = Hemoglobin; HF = Heart Failure; HR = Heart Rate; IADLs = Instrumental Activities of Daily Living; ISI = Insomnia Severity Index; MMSE = Mini-Mental State Examination; MOS-SSS = Medical Outcomes Study Social Support Survey; MUST = Malnutrition Universal Screening Tool; NYHA Class = New York Heart Association Functional Classification; SBP = Systolic Blood Pressure; SCr = Serum Creatinine; SUA = Serum Uric Acid; SE = Standard Error; TC = Total Cholesterol

| **Table S3.** Spearman’s correlation matrix for continuous variables retained from the univariable analysis. | | | |
| --- | --- | --- | --- |
| Variable Pair | ρ | 95% CI | *P*-value |
| IADLs- Rating of mobility | 0.068 | -0.039, 0.174 | 0.213 |
| IADLs- MOS-SSS | 0.020 | -0.088, 0.126 | 0.721 |
| Rating of mobility- MOS-SSS | 0.032 | -0.075, 0.139 | 0.557 |

Note:

Abbreviations: ρ = correlation coefficient; CI = Confidence Interval; IADLs = Instrumental Activities of Daily Living; MOS-SSS = Medical Outcomes Study Social Support Survey; SE = Standard Error
